# Establishment, optimization, and application of genetic technology in *Aspergillus* spp.

**DOI:** 10.3389/fmicb.2023.1141869

**Published:** 2023-03-21

**Authors:** Jing Gao, Huiqing Liu, Zhenzhen Zhang, Zhihong Liang

**Affiliations:** ^1^College of Food Science and Nutritional Engineering, China Agricultural University, Beijing, China; ^2^The Supervision, Inspection and Testing Center of Genetically Modified Organisms, Ministry of Agriculture, Beijing, China; ^3^Beijing Laboratory for Food Quality and Safety, College of Food Science and Nutritional Engineering, China Agricultural University, Beijing, China

**Keywords:** genetic engineering, homologous recombination, CRISPR, RNA interference, transformation, selective marker, cell factory

## Abstract

*Aspergillus* is widely distributed in nature and occupies a crucial ecological niche, which has complex and diverse metabolic pathways and can produce a variety of metabolites. With the deepening of genomics exploration, more *Aspergillus* genomic informations have been elucidated, which not only help us understand the basic mechanism of various life activities, but also further realize the ideal functional transformation. Available genetic engineering tools include homologous recombinant systems, specific nuclease based systems, and RNA techniques, combined with transformation methods, and screening based on selective labeling. Precise editing of target genes can not only prevent and control the production of mycotoxin pollutants, but also realize the construction of economical and efficient fungal cell factories. This paper reviewed the establishment and optimization process of genome technologies, hoping to provide the theoretical basis of experiments, and summarized the recent progress and application in genetic technology, analyzes the challenges and the possibility of future development with regard to *Aspergillus*.

## 1. Introduction

The discovery of the DNA double helix in 1953 is considered the beginning of molecular genetics (Wilkins et al., 1953). Since Sanger (Sanger et al., 1977) invented the dideoxy termination method (also named chain termination method) as well as Maxam and Gilbert (1977) invented the chemical degradation method to sequence genes, humans have obtained the key to the genome. At present, genome sequencing technology has developed to the third generation of high-throughput single-molecule sequencing, which facilitates us to quickly obtain genomic data of different species (Chiara et al., 2021). Subsequently, the principles of gene damage and repair have been clarified that provided a theoretical basis for artificially modified genes. Finally, the discovery of various restriction enzymes and ligases, which are known as “molecular scissors and glues,” provides powerful tools. Gene editing technologies came into being, realized the replacement, insertion, deletion, and modification of specific nucleic acid segments (Pant et al., 2022).

**Figure 1:**
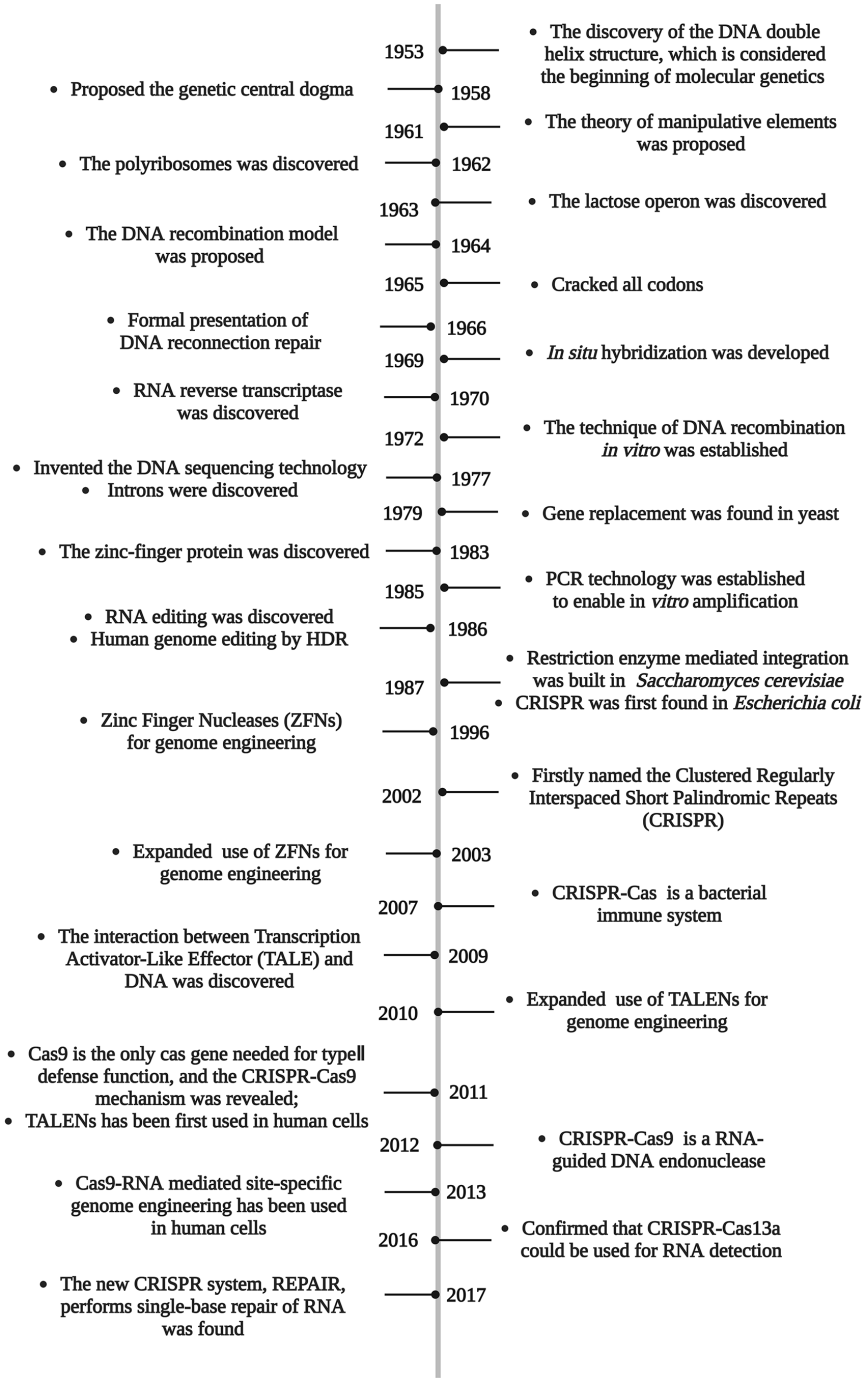
The history of gene editing technology.

Scientists have long attempted to acquire new traits that do not exist in nature but meet human expectation by influencing genes of organisms. The simplest and most crude way to induce mutations is through physicochemical methods, including physical mutagens (such as UV light, ionizing radiation, and radioactive material) and chemical mutagens (such as benzene and alkaloid; Rivera et al., 2014), but extreme uncertainty and tedious laborious works limit their systematic-scale use. Molecular tools are then used to create random genetic mutations through the insertion of foreign nucleic acid segments or the movement of mobile genetic elements. Since random mutation has the potential risk to disrupt or alter other genes, new strategies have been explored to solve this uncontrollability. Finally, specific and targeted technologies combined with the homologous recombination mechanism realize the current arbitrary study of target genes (Li et al., 2020). From the initial simple gene replacement in yeast in 1979 (Struhl et al., 1979) to the advent of multifunctional Clustered Regularly Interspaced Short Palindromic Repeats (CRISPR)-Cas9 technology that is currently popular around the world (Jinek et al., 2012), gene editing technologies have been explored for more than 40 years (Figure 1). Genetic engineering can break through species limits, which is expected to achieve unprecedented scientific breakthroughs, but in order to avoid genetic contamination in nature and to ensure ethical compliance, some legal framework on genetically modified organism or strain (GMO) have been enacted, such as the Directive 2001/18/EC of the European Parliament, that genetic engineering must be carried out under strict laws (Garrigues et al., 2021). Recent advances in genome biology have expanded the field of genetic engineering, various methods can be used alone or in combination to achieve unprecedented collaboration and for interdisciplinary applications.

*Aspergillus* is a class of saprophytic filamentous fungi that reproduce by asexual conidiospores and sexual ascospores, this genus contains four subgenera and 339 species, and are distributed ubiquitously in natural habitats (Houbraken et al., 2020). Some *Aspergillus* species, such as *A*. *nidulans*, *A*. *niger*, and *A*. *oryzae* are Generally Recognized As Safe (GRAS) by the Food and Drug Administration (FDA), playing significant roles in industrial biotechnology and fermentation technology, and as extraordinary eukaryotic host for microbial cell factories for the production of enzymes, antibiotics, nutrilite, and so on (Li et al., 2022). However, some *Aspergillus* species are terrible food spoilage fungus, which widely contaminating raw materials and processed foods, spatiotemporally involving planting, harvesting, processing, storage and sales, causing foods sensory quality reduction, physical damage, and chemical composition destruction (Luo et al., 2018). These fungi can also produce harmful secondary metabolites mycotoxins. The Food and Agriculture Organization (FAO) has reported 25% contamination of food by mycotoxins worldwide (Eskola et al., 2020), and most mycotoxins are certified by the International Agency for Research on Cancer (IARC) as Group I or Group II carcinogens, which pose a serious threat to animals and humans health (Ostry et al., 2017).

In recent years, with the popularization of gene sequencing technology, genome sequences of filamentous fungi have been established (http://www.genomesonline.org/; http://genome.jgi.doe.gov/), and the explosive growth of *Aspergillus* whole genomes data provide the possibility to change fungal characters (Robey et al., 2021). The gene expression can be controlled during both transcription and post transcription levels through gene editing technologies, then combined with omics analysis and high-throughput screening technologies has achieved industrial application. In this review, we systematically summarized the genetic modifications in *Aspergillus*, from the development process, gene editing techniques, to efficient transformation methods, and hoping to facilitate the background supplement of experimenters. Furthermore, we discuss the major challenges and solutions of genetic engineering, and finally, we propose potential directions in the development of the *Aspergillus* system, which would be helpful for the research and development of *Aspergillus* expression cell factories or mycotoxin prevention and control, and then draw on advantages and avoid disadvantages.

## 2. Genome editing technologies

### 2.1. Homologous recombination based systems

Homologous Recombination (HR) based systems are relatively early gene editing technologies and a breakthrough in eukaryotic gene editing (Capecchi, 1989). The principle is to introduce the foreign gene into the recipient cell, and the exogenous DNA fragment replaces the target gene *in situ* through site-specific recombinases mediating the recombination of target sites to achieve specific gene modification. Typical examples include the Lambda-red system, which is only for *Escherichia coli*, as well as the Cre-*lox*P system in *Saccharomyces cerevisiae* and the similar FLP-FRT system that are widely applicable to fungi.

#### 2.1.1. Lambda-red system

In 1998, Murphy reported the use of λ bacteriophage Red system (Lambda-red system) for gene modification in *E*. *coli*, which was an early implementation of sequence-specific recombinases (Murphy, 1998). It does not rely on the recombinant system of *E*. *coli* own, but uses three proteins (Exo, Gam, and Beta) from bacteriophages through vector plasmids transformation to modify genes. Exo exonuclease degrades linear double-stranded DNA (dsDNA) starting from the 5′ ends to produce single-stranded DNA (ssDNA) fragments with a 3′ sticky end, Gam protein prevents the *E*. *coli* endogenous RecBCD and SbcCD nucleases from cutting linear DNA, Beta annealing protein protects the ssDNA created by Exo (Dillingham and Kowalczykowski, 2008). The Lambda-red system is independent of restriction sites and has applied to gene editing flexibly, but it is only suitable for *E*. *coli* (Zeng et al., 2022). However, this system in *E*. *coli* can be used to achieve efficient expression of fusion PCR fragments and based on homologous recombination to achieve gene editing in *Aspergillus* (Chaveroche et al., 2000; Langfelder et al., 2002). In addition, with the development of new technologies, the Lambda-red system can already be more widely used by combining with such as CRISPR technology.

#### 2.1.2. Cre-*lox*P and FLP-FRT system

**Figure 2:**
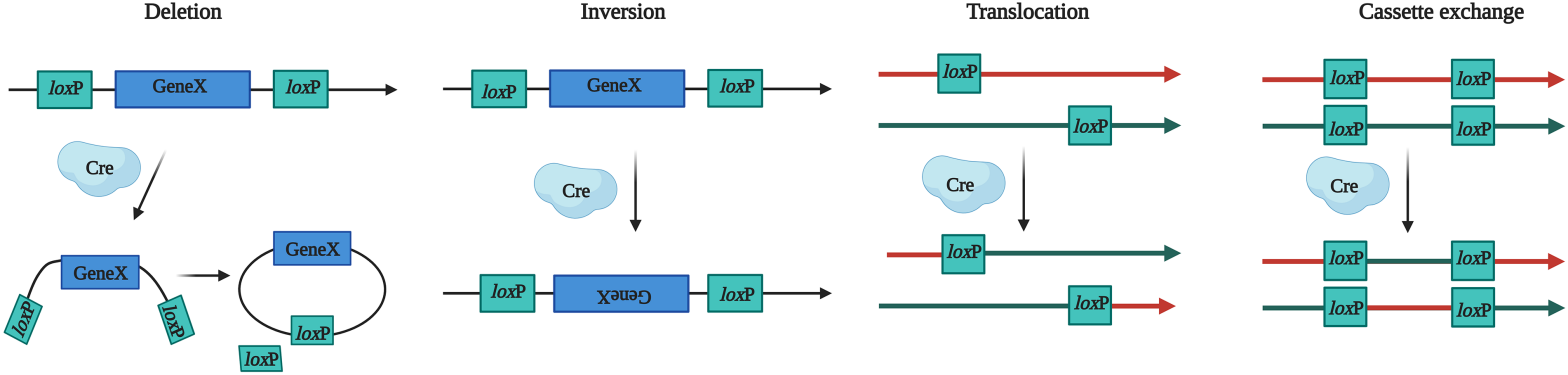
Biological mechanisms for Cre–*lox*P system. Cre recombinase can recognize the *lox*P site and promote gene recombination. *Lox*P is a unique gene sequence from the P1 phage, which guides the Cre to target sites of the genome. Gene X stands for target gene. The Cre–*lox*P system can produce four types of gene modification, including deletion, inversion, translocation, and cassette exchange.

In 1981 (Sternberg and Hamilton, 1981), the DNA sequence specific Cyclization recombination enzyme (Cre) was discovered in P1 bacteriophages, and its C-terminal domain contains catalytic active sites that can catalyze recombination between pairs of locus of X-overP1 (*lox*P) sites. The *lox*P site consists of two reverse palindromic sequence domains and the intermediate spacer sequence, the former identifies and binds the Cre and the latter determines the direction of *lox*P sites. Cre recombinase can act on DNA substrates of various structures (such as linear, circular, and supercoiled DNA) independent of cofactors. Depending on the location and orientation of *lox*P sites in the genome, Cre recombinase can initiate deletions, inversions, translocations, and cassette exchange of the two *lox*P sites that flank a genomic segment of interest (named “floxed” locus; Nagy, 2000; Figure 2).

The Cre–*lox*P system provides the possibility for multigene manipulation that need two steps to achieve gene recycling, *lox*P sites and a marker gene need to be integrated into the host genome firstly, then the Cre was expressed to complete the gene modification process. The Cre–*lox*P system has been successfully applied in *Aspergillus* spp. Modified Cre-*lox*P system is a powerful tool for producing high levels of various organic acids in *A*. *niger* (Xu et al., 2019), and this site-specific recombinase implemented the integration of a heterologous DNA fragment as large as 21 kb in *A*. *nidulans* (Roux and Chooi, 2022). In addition, Cre recombinase has been used in *Saccharomyces* (Moon et al., 2022), *Lecanicillium* (Nguyen et al., 2018), *Trichoderma* (Steiger et al., 2011), *Neurospora* (Honda and Selker, 2009), *Neotyphodium* (Florea et al., 2012) and so on, setting the foundations for the further development of fungal cell factories.

The FLP-FRT system derived from *S*. *cerevisiae* is similar to the Cre-*lox*P system (Sadowski, 1995), using flippase (FLP) recognizes a pair of FLP recombinase target (FRT) sequences that flank a genomic region of interest, and also rely on HR with 30–50 base-pairs of homologous arms for non-specificity gene knockin or knockout. The FLP-FRT system was first successfully applied in *Penicillium chrysogenum* and *Sordaria macrospora* (Kopke et al., 2010). At present, these techniques have developed into recombination-based genetic engineering (recombineering), which widely applied in yeast, filamentous fungi, plant, and mammal (Zeng et al., 2022), However, it can only act on specific sequences in the genome that are recognized by recombinases, and there is a high probability of misintegration.

### 2.2. Sequence specific nuclease based technologies

**Figure 3:**
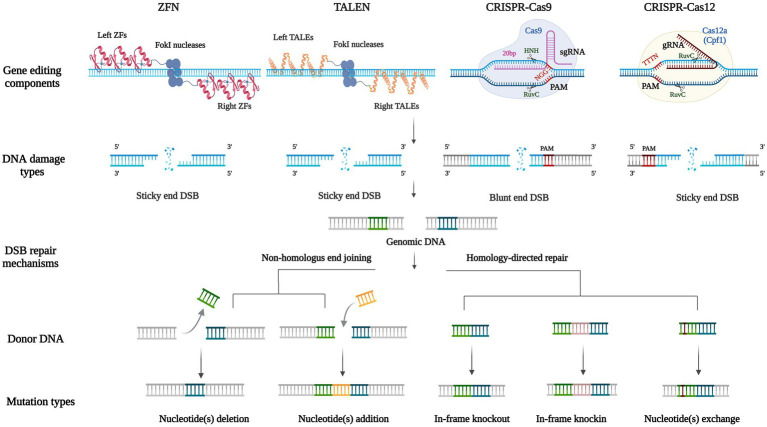
Biological mechanisms for nuclease-based gene editing technologies. Including gene editing components, the type of DNA damage, double-strand break repair mechanisms, donor DNA, and mutation types of ZFN, TALEN, CRISPR-Cas9, and CRISPR-Cas12 systems. Both ZFN and TALEN systems locate similar FokI endonucleases (the dark blue combined sphere) by DNA-binding domains on the left and right of the target site (the red zinc finger domains and the yellow transcription activator-like effectors domains), producing sticky ends double-strand breaks. The CRISPR systems consist of guiding RNA to locate target fragments containing PAM (the bases highlighted in red), and Cas nuclease domains cleave DNA to produce double-strand breaks. Cas9 includes HNH and RuvC catalytic nuclease domains, each cleaving one strand of the target DNA to produce a blunt end. Cas12 contains a RuvC nuclease domain that acts on both single strands of the target DNA to produce a sticky end. NHEJ and HDR are two DSB repair mechanisms, based on the complementarity of homologous fragments between genomic DNA and donor DNA (base pairs of the same color), to produce different mutation types including nucleotide(s) deletion, nucleotide(s) addition, in-frame deletion, in-frame knockout, and nucleotide(s) exchange.

Compared with traditional HR gene editing technologies, nuclease-based gene editing techniques are also rely on homologous sequences. However, designed nucleases can more accurately act on target sequences to reduce random insertion, and more importantly, enable realize the recognition and modification of arbitrary gene sequences. Currently, manipulation nuclease-based gene editing technologies include Zinc Finger Nuclease (ZFN) system, Transcription Activator-Like Effector Nuclease (TALEN) system, Clustered Regulatory Interspaced Short Palindromic Repeat-CRISPR associated protein (CRISPR-Cas) system and single Base Editor (BE), etc. The first three systems are based on the genomic target site causing Double-Strand Breaks (DSBs) of DNA, which in turn activates the internal repair mechanism of cells. Among them, CRISPR has emerged as a powerful instrument for exploring genomics (Pant et al., 2022). The principles and comparison of these three techniques are shown in charts (Figure 3; Table 1). In 2016, the development of BE technology realized single base conversion without causing DSBs of DNA and no more need for homologous templates, effectively avoiding the off-target effect (Komor et al., 2016).

**Table 1:** Systematic comparison of nuclease-based gene editing technologies.

Gene editing technology	Zinc finger nuclease (ZFN)	Transcription activator-like effector nuclease (TALEN)	Clustered regularly interspaced short palindromic repeat-CRISPR associated protein (CRISPR-Cas9)
Occurrence	1996	2009	2012
Origin	Eukaryotic, prevalent in almost all eukaryotes	Eukaryotic, from *Xanthomonas*	Prokaryotic, from *Streotococcus pyrogens*
Target sequence	Zinc Finger (ZF) domains	Repeat variable diresidues (RVDs).	crRNA/sgRNA
Effector molecule	FokI	FokI	Cas9
Length of target sequence	(3–6) × 3 bp × 2	(8–31 bp) × 2	20 bp + 5’-NGG-3’ PAM sequence
Cytotoxicity	Severe	Minor	Least
Design of construction	The most difficult	Medium difficulty	Easy
Degree of methylation sensitivity	Sensitive	Sensitive	Insensitive
Off-target effects	Highest	Low	Higher
Off-target detection	Low predictability	Low predictability	High predictability
Efficiency of cutting	High	Higher	Highest
Limitations	Not suitable for high-throughput targeting of target genes; Difficult design, instability, high off-target rate	Require a 5′-thymine base in the target site; Repetitive sequences cause nonspecific cleavage	Require PAM motifs adjacent to the target site; The off-target rate is relatively high

#### 2.2.1. Zinc finger nuclease

In 1996, scientists introduced the Zinc Finger Nuclease (ZFN) technology (Kim et al., 1996). ZFN monomer is a protein fused with a non-specific FokI endonuclease catalytic domain and 3–6 Cys2-His2 Zinc Finger (ZF) DNA-binding domains, each of which targets a DNA triplet base pairs. The dimerization of two FokI nuclease domains, by which designing ZF domains in both directions of 5′–3′ and 3′–5′ for target sequences and connecting them to different Fok1 respectively, then the middle target DNA sequence could be cleaved into DSBs (Carroll, 2011; Figure 3). The application of ZFN in *A*. *nidulans* is relatively mature (Fayyaz et al., 2020), but still not been widely used due to the cumbersome process of constructing ZF domains and verifying their specificity, but also because this system has some cytotoxicity, and thus there have few studies in fungi.

#### 2.2.2. Transcription activator-like effector nuclease

Transcription Activator-Like Effector (TALE) was first found in the plant pathogen *Xanthomonas* in 2009 (Boch et al., 2009). Each TALE contains a central region of tandem direct repeats composed of 33–35 amino acids (mostly 34), this sequence is conserved except for the 12 and 13th amino acid residues, which are named Repeat Variable Diresidue (RVD). The 12th amino acid residue stabilizes the RVD loop, whereas the 13th amino acid residue makes a base-specific contact in the target DNA bases (NI recognizes A, NG recognizes T, HD recognizes C, and NN recognizes G) and therefore TALENs are able to identify Single Nucleotide Polymorphisms (SNPs; Bedell et al., 2012; Figure 3). The composition of TALENs is similar to ZFNs in that their carboxylate terminal are both fused with a catalytic FokI endonuclease to produce a specific DSB. However, compared with the triplet base pairs recognized by each ZFN monomer, one TALEN monomer recognizes only one base and therefore a specific TALEN nuclease can theoretically be designed and constructed for any DNA sequence, to improve its specificity and flexibility (Cermak et al., 2011; Joung and Sander, 2013). At present, TALEN technology has been successfully applied to generate relatively stable genetic modification of human cells and model organisms including *Drosophila*, *Caenorhabditis elegans*, zebrafish, mice, and *Arabidopsis thaliana* (Fayyaz et al., 2020). In *A*. *oryzae*, it has been verified that the TALEN can achieve the deletion of large fragments of the target sequence (Mizutani et al., 2017). However, each base of the target sequence requires a TALE recognition module, so the construction process of TALENs still laborious.

#### 2.2.3. Clustered regularly interspaced short palindromic repeats-CRISPR associated protein system

In 1987, Ishino et al. discovered the repeated sequences of palindromic repeats interspaced by a 32 bp sequence in *E*. *coli* (Ishino et al., 1987), and Dutch scientists Jansen et al. first named it the CRISPR and CRISPR-associated protein (Cas; Jansen et al., 2002). Subsequently, Barrangou et al. demonstrated that CRISPR-Cas is an adaptive immunity system present in bacteria and archaea (Barrangou et al., 2007). The *in vitro* remodeling of CRISPR-Cas9 (Jinek et al., 2012) and the demonstration of its gene editing function in human cells (Cong et al., 2013) marked the beginning of a new era of gene engineering. In 2015, following the successful genome editing of the CRISPR-Cas9 system in the model fungus *N*. *crassa*, Mortensen’s team established a universal CRISPR-Cas9 system for the first time in six species of *Aspergillus* (*A*. *nidulans*, *A*. *niger*, *A*. *aculeatus*, *A*. *brasiliensis*, *A*. *carbonarius*, and *A*. *luchuensis*; Nodvig et al., 2015).

##### 2.2.3.1. Mechanism of CRISPR-Cas system

According to the complex of Cas proteins and the domains organization of the effectors, CRISPR-Cas systems are divided into two classes including six different types. Class 1 includes Type I, Type III, and Type IV, and need the Cas complex participating in the recognition and degradation of foreign DNA. Class 2 contains three types (II, V, and VI) and divided into 10 subtypes, which only need one Cas protein mediated by RNA to perform interference function, has been greatly developed in gene editing (Haft et al., 2005; Koonin et al., 2017).

**Figure 4:**
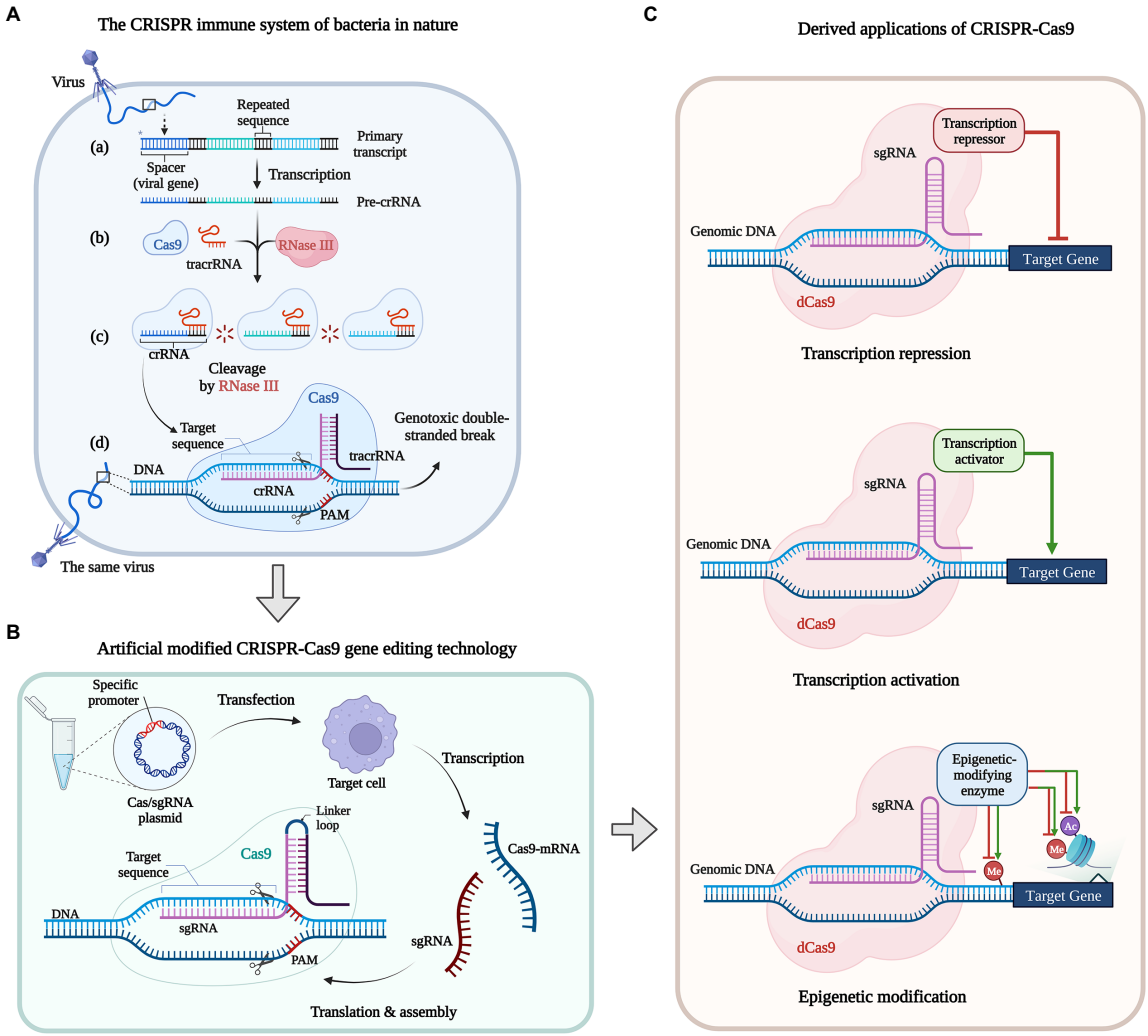
Development of the CRISPR-Cas system: natural, modified, and derived applications. **(A)** The CRISPR immune system of bacteria in nature. **(a)** Viral DNA is inserted into the bacterial genome as a spacer in between the repeated CRISPR sequences. **(b)** Trans-activating RNA (tracrRNA) recognize CRISPR-RNA (crRNA) sequences and target Cas9 enzymes to the crRNA. **(c)** The crRNA: tracrRNA duplex cleave the longer pre-crRNA by RNaseIII and then produce mature crRNA. **(d)** When the same virus infect bacterium again, the Cas9: crRNA generated during the first infection recognizes Protospacer Adjacent Motif (PAM) motifs in the viral genome, enabling target-DNA recognition and Cas9-mediated cleavage to prevent the re-infection. **(B)** Artificial modified CRISPR-Cas9 gene editing technology. *In vitro* designed Cas/ single guide RNA (sgRNA) expression plasmid(s) with specific promoters are transduced into target cells, Cas/sgRNA mRNAs are transcribed *in vivo*, which are translated, modified, and assembled to form a CRISPR-Cas9 system. **(C)** Derived applications of CRISPR-Cas9. dead Cas9(dCas9) can be used to mediate transcriptional repression, activation and epigenetic modification of target genes when fused with a small-molecule repressor, activator and epigenetic-modifying enzymes.

The Class 2 Type II CRISPR-Cas9 systems have been studied relatively thoroughly. In the natural bacterial CRISPR-Cas9 system, the Cas9 contains two single stranded nuclease domains, RuvC (the crossover junction endodeoxyribonuclease) and HNH (named for characteristic histidine and asparagine residues). The guide RNA (gRNA, i.e., protospacer) exists as a CRISPR array, transcribed to form a primary transcript, and then processed into mature CRISPR-RNA (crRNA) with the participation of RNase III. Parts of infective viruses or phages DNA sequence would be integrated into the bacterial CRISPR array. When the foreign DNA invades again the mature crRNA combined with a trans-activating RNA (tracrRNA) to form crRNA: tracrRNA dimers, which can combine Cas9 protein and lead nucleases to degrade this foreign DNA based on complementary sequences (Barrangou et al., 2007; Jiang and Doudna, 2017; Figure 4A).

Currently, the modified CRISPR-Cas9 system derived from *Streptococcus pyogenesis* is the most mature type of CRISPR-Cas (Cong et al., 2013), and *S*. *cerevisiae* is the first fungal species that had implemented CRISPR-Cas9 for genome editing (DiCarlo et al., 2013). Adding a linker loop between crRNA and tracrRNA fuses the crRNA: tracrRNA dimers into a more refined single guide RNA (sgRNA), which composed of two parts (Pant et al., 2022). The 20-bp gRNA part responsible for identifying genomic DNA regions with a short Protospacer Adjacent Motif (PAM) composed of three bases of NGG (N stands for any base), and the gRNA scaffold part binding to Cas9 and activate its ability of cleavage (Jinek et al., 2012). The target strand hybridizing to gRNA and the complementary strand is, respectively, cleaved by HNH and RuvC then produce a blunt end DSB at the target region which is located 3–5 bases upstream of the PAM (Hsu et al., 2014; Figure 4B).

The Class 2 Type V effector Cas12a, also called Cpf1 (Cas in *Prevotella* and *Francisella 1*), requires no tracrRNA, which uses the guide crRNA alone to target dsDNA and activates RuvC to cut both strands of the target DNA then produce a sticky end DSB (Zetsche et al., 2015). Vanegas et al. first realized the application of Cpf1 in *A*. *nidulans*, which catalyzed oligonucleotide-mediated genomic site directed mutagenesis and marker-free gene targeting (Vanegas et al., 2019). The crRNA of Cas12a is significantly shorter and more flexible than Cas9 sgRNA, thus thought that Cas12a has the ability to target multiple genome loci simultaneously, as well as possess RNase activity in addition to its DNA cleavage activity. In addition, the new Class 2 Type VI effector Cas13 that can edit RNA, has considerable research value (Li et al., 2019; Safari et al., 2019).

##### 2.2.3.2. Application of CRISPR-Cas system

Clustered Regularly Interspaced Short Palindromic Repeats-Cas systems provide diversified sequence-specific gene regulation tools that have led to various gene editing techniques. Cas9 nuclease becomes the Cas9 nickase (Cas9n) when inactivate the HNH or RuvC nuclease domain that induces single-strand break (also called nick) instead of DSB, but do not interfere with the binding and interaction of gRNA with the target DNA double strands. Thus CRISPR-Cas systems can be modified for diverse functions based on the nuclease deactivated Cas9 (dCas9) combined with regulatory parts (Figure 4C; Kundert et al., 2019; Uygun et al., 2020). For example, the CRISPR activation (CRISPRa) is fusing a transcriptional activator or multiple copies of the activation domain, while CRISPR interference (CRISPRi) is fusing repressor domains to the Cas9n enzyme that can block the elongation of transcripts (Qi et al., 2013). Epigenetic modifications at specific genomic locations can be achieved when fusing dCas9 with epigenetic-modifying enzymes, for example, the modification of methyl or acetyl groups for the histidine of target gene can affect gene function to a certain extent (Zhuo et al., 2021). Moreover, multiple sgRNA expression plasmids can be transferred into one cell to edit multiple genes synchronous, such as *A*. *oryzae* (Li et al., 2023) and even have the potential function of genome screening (Cong et al., 2013; Zetsche et al., 2017; Zeng et al., 2022). At present, a considerable part of common *Aspergillus* gene function studies have applied the CRISPR technology, also have applied in fungal factories, such as glucoamylase-hyperproducing industrial in *A*. *niger* (Liu D. et al., 2022) and kojic acid production in *A*. *oryzae* (Chen Z. et al., 2022).

##### 2.2.3.3. Improvement of CRISPR-Cas system

Compared with ZFN and TALEN, CRISPR-Cas improved the accuracy of gene editing by gRNA matched to the target DNA region. In addition, unlike the FokI endonuclease must be dimerized to cleave targeted DNA, Cas performs functions as a monomeric protein thus avoiding delicate and complex protein design and assembly. However, Cas nucleases may inherit the low sequence specificity from the prokaryotic innate defense system, which increases the chance of non-specific cleavage and off-target effect.

Many strategies for components efficient expression, normal operation and function expansion have been developed. The operation of the CRISPR-Cas system requires the presence of two key component elements in hosts, Cas protein and sgRNA, Zheng et al. summarized three types of transformation in *A*. *niger*: (1) Cas protein expression plasmid and sgRNA expression plasmid both in DNA form, (2) sgRNA in RNA form and Cas protein expression plasmid in DNA form, and (3) RNP complex (Cas/sgRNA ribonucleoprotein complex) formed *in vitro*, which provides a useful reference for the application of CRISPR technology in *Aspergillus* (Zheng et al., 2021). The construction of Cas protein and sgRNA expression cassette is crucial, and the selection of promoters is related to the efficient expression of component elements and the successful application in target organisms. The promoters used for Cas protein expression are mostly constitutive strong promoters such as P*gpdA* from *A*. *nidulans* (Zhang et al., 2016), P*tef1* (Nodvig et al., 2015), P*coxA* (Sarkari et al., 2017), and *pkiA* (Song et al., 2018), or inducible promoters such as P*glaA* from *A*. *niger* (Zhang et al., 2020). Fusing a nucleus localization signal on the plasmid can precisely translocate the Cas9 protein to the nucleus (Goeckel et al., 2019). The gRNA expression cassettes are divided into promoters recognized by RNA polymerase II or RNA polymerase III. The use of pol II promoter, including *gpdA*, *mbfA*, etc., requires the presence of ribosomal splicing sequence for pre-crRNA processing. The pol III promoter such as U6 (Zhang et al., 2016), the *S*. *cerevisiae* SNR52 promoter (Fuller et al., 2015), and the bacteriophage T7 promoter (Kuivanen et al., 2016), have been optimized for high transcription. Various tRNA promoters can also drive the sgRNA expression but the expression level is low (Song et al., 2018), and the endogenous 5S rRNA promoter proposed by Zheng et al. can significantly improve the expression level of sgRNA and achieve 100% gene targeting inactivation efficiency (Zheng et al., 2019). More professional and detailed description has been reviewed by Zheng et al. (2021) and references therein. Adding a cell-specific promoter to the Cas or sgRNA expression vector can achieve the spatiotemporal control of gene editing, and counteracts undesired side effects in non-target cells (Zhou et al., 2018; Maggio et al., 2020). Other studies showed that both Cas9 and Cas12a proteins can be engineered to recognize different PAM sequences thus expanded the application scope (Corsi et al., 2022; Zhu et al., 2022). Kleinstiver et al. broadened the targeting range of *Staphylococcus aureus* Cas9 by modifying PAM sites in human cells, although the specificity and off-target effect remains not very ideal (Kleinstiver et al., 2015a,b).

Improvements in the high off-target rate of CRISPR can be achieved by modifying the components. One approach is to design truncated guide sequences, which minimize mismatches and secondary structures of sgRNA that reduce off-target binding (Fu et al., 2014). Modifications directed to control active Cas amount can reduce unnecessary and non-specific cleavage, some physicochemical methods such as light (Nihongaki et al., 2015) or exogenous small molecules (Dow et al., 2015) can partly inhibit the Cas9 expression. Another way is to use the Cas nickase instead of the Cas nuclease, producing the single-strand break instead of the double-strand break, which does not induce NHEJ repair and still activates precise HDR repair (Kuo et al., 2020). Single-strand breaks can reduce off-target effects, but also reduce repair efficiency, so it has been proposed to use double nickases, such as two Cas9ns (Ran et al., 2013), two cpf1n (Suzuki et al., 2016; Zetsche et al., 2017), and dCas9-FokI dimers (Tsai et al., 2014), to improve the specificity and efficiency of gene editing.

Due to its simplicity, operability, and efficiency, CRISPR has been widely used in a variety of advanced eukaryotes that are difficult to operate with traditional gene editing strategies. Meanwhile, several laboratories around the world have developed various CRISPR-Cas systems for *Aspergillus* (Nakamura et al., 2021). Recently studied in *A*. *flavus*, the CRISPR-Cas9 utilized the autonomous maintenance in *Aspergillus* (AMA1) autonomously replicating sequence has achieved relatively efficient multiple-gene knockouts, satisfactory gene-targeting efficiencies (>90%) have also been obtained in *A*. *nidulans*, *A*. *fumigatus*, *A*. *terreus*, and *A*. *niger* (Chang, 2023). It likely have a broad application in aspergilli.

#### 2.2.4. Base editor

Zinc Finger Nuclease, TALEN, and CRISPR-Cas all rely on Non-Homologous End Joining (NHEJ) and Homology Directed Repair (HDR), which induce breaks at target sites to activate DNA repair. NHEJ is prone to cause frameshift mutations, which in turn affect the function of target genes. Although the accuracy of HDR is higher than NHEJ, its efficiency in cells is very low, only about 0.1–5%. The Base Editor (BE) technology does not require DSBs or homologous templates to perform single base conversion, which has effectively improved the above problems (Komor et al., 2016). BE technique combines nuclease-free dCas9 or Cas9n, cytosine deaminase, DNA Uracil Glycosylase Inhibitor (UGI), and sgRNA. It is possible to directly deaminates cytosine (C) to uracil (U) at the targeted site without DSBs, and the U is not excised due to the presence of uracil glycosylase inhibitor. As DNA replicates, U is replaced by thymine (T), and guanine (G) that was originally complementary to C will be replaced with adenine (A), finally achieving single-base precise editing of C to T and G to A within a certain active frame (Gaudelli et al., 2017; Chen L. et al., 2022). The advent of BE technology has promoted the effectiveness and scope of use of point mutation gene editing. If the termination codon (TAA, TGA, and TAG) appears in advance, gene knockout can be achieved. For multi-copy sites, Cas9 will produce multiple DSBs, causing DNA damage and even apoptosis, so under the same conditions, BE-induced terminator generation to achieve gene knockout is safer than Cas, and can be used as a safe and efficient genome-wide screening system (Chen L. et al., 2022; Liu N. et al., 2022).

### 2.3. RNA technologies

Some RNA technologies, including RNA interference (RNAi) and ribozymes, can verify gene function through disturbing mRNA translation have also applied in fungi.

**Figure 5:**
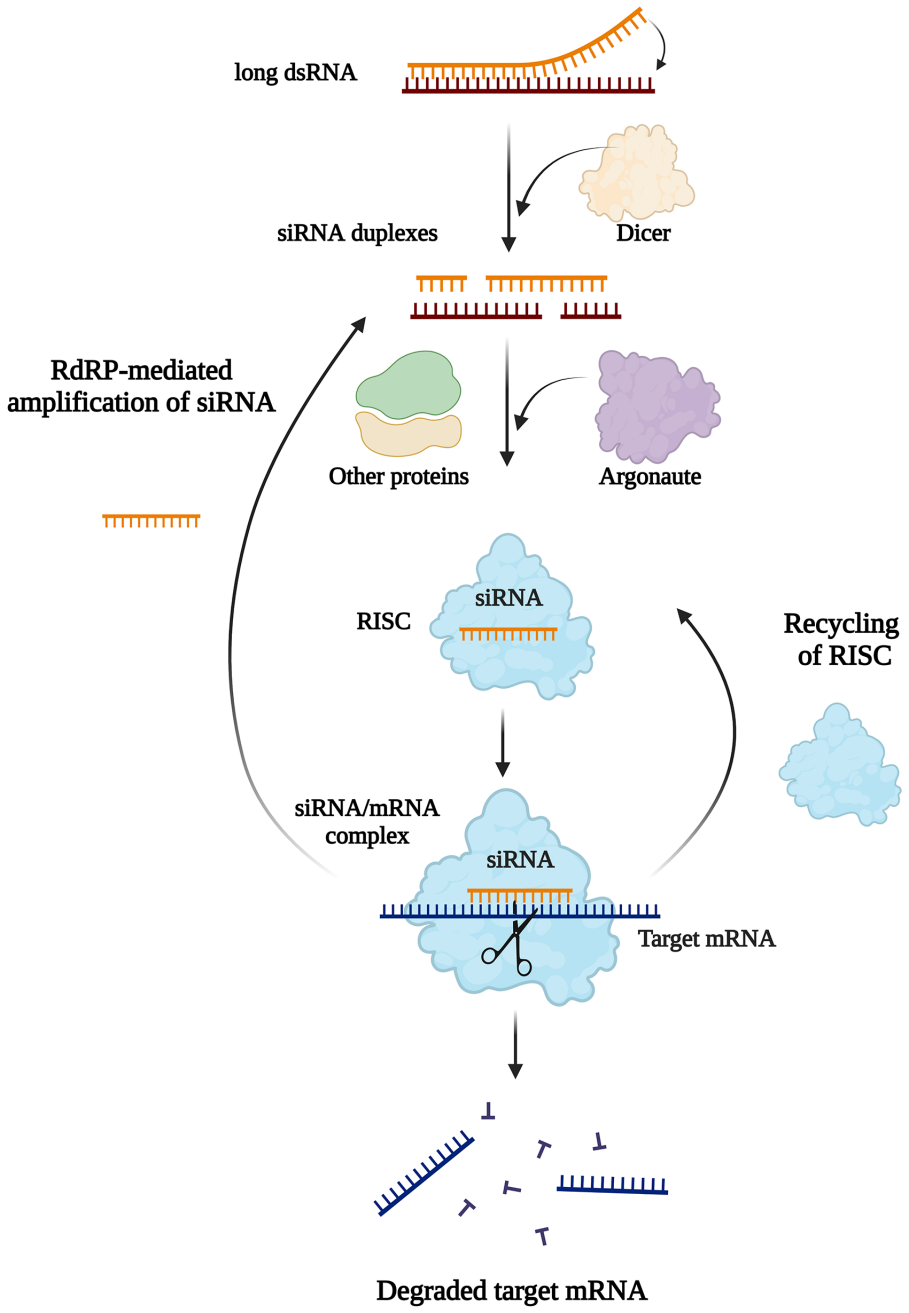
Biological mechanisms of RNAi-mediated gene silencing. Long double-stranded RNAs or hairpin RNAs bind to Dicer are cleaved, and then generate multiple 21–23 bp double-stranded small interfering RNAs (siRNAs) duplexes. Each siRNA is unchained into a passenger (sense) strand and a guide (antisense) strand by intracellular RNA helicase. The guide strand binds with Argonaute (AGO) and other proteins to form an RNA Induced Silencing Complex (RISC), while the passenger strand is degraded by subsequent cellular events in the cytoplasm. The siRNA/RISC complex then binds the complementary mRNA resulting in the degradation of the target transcript or inhibition of translation. The components of siRNA/mRNA complex can be recycled to the RISC complex or generate siRNA duplexes by the action of RNA-dependent RNA Polymerase (RdRP).

RNA interference (RNAi) evolved by eukaryotes is a conserved transcriptional and post-transcriptional levels gene silencing mechanism, which is involved in multiple biological processes, particularly in host defense and gene regulation (Fire et al., 1998). The filamentous fungus *Neurospora crassa* is one of the first organisms used for RNAi studies in 1992 (Romano and Macino, 1992). RNAi is adopted by inactivating RNA translation *via* the RNase III enzyme (Dicer) in an ATP-dependent manner without affecting the normal gene expression (Fire et al., 1998; Borges and Martienssen, 2015; Nargesi et al., 2021; Figure 5). It should be noted that due to the high specificity and potential efficiency of RNAi inhibitory gene expression, a relatively less amount of dsRNA molecules (far less than the amount of endogenous mRNA) can completely inhibit the expression of the corresponding gene, which carried out in a catalytic scale-up and may cause needless and even harmful gene silencing. Therefore, the effective expression of various genes in normal body has a set of mechanisms to strictly prevent the formation of dsRNA (Liu et al., 2021; Wang et al., 2021). The RNAi technology has been used in many higher eukaryotes by transferring designed siRNA into the host for gene knockdown. Methods for siRNA preparation include chemical synthesis, *in vitro* transcription, degradation of long dsRNA by RNase III, expression of siRNA in cells through siRNA expression vector or viral vector, and siRNA expression frame prepared by PCR (Gebremichael et al., 2021). In summary, RNA silence has the potential to offer efficient tools that gene disruption methods cannot provide in the exploration of gene function, but there are also shortcomings (Table 2). Detailed analyses were carried out by Abo-Al-Ela (2021) and Gebremichael et al. (2021).

**Table 2:** Systematic comparison of DNA disruption and RNA interference.

	DNA disruption	RNA interference
Mechanism	Repair of DNA breaks after cleavage by nucleases	Gene silencing at the post-transcriptional level
Target	Genome specific region	mRNA specific region
Mode of manner	Locus-specific	Sequence-specific
Mutation type	Knockout& Knockin& Nucleotide(s) exchange	knockdown
Gene suppression	Complete	Partial
Phenotypic impact	Definite and complete	In varying degrees
Recovery of mutation	Genetic complementation	Resurrection gene with synonymous substitutions
Limitations	Knockout of vital genes may be fatal; Unable to perform gene expression in specific spatiotemporal progress; Multigene knockout is restricted.	Mistakenly silence other genes with high homology to the target gene； Unable to completely revive the knocked down gene and apply it to a large-scale experiment.

In 1980, the ribozyme (also known as catalytic RNA) was found to have the specific catalytic function similar to enzyme, which expands the range of enzymes from proteins to nucleic acids. Ribozymes can specifically recognize and cut target mRNAs then detach from degraded mRNAs and bind to other target mRNAs without affecting the host RNA. Ribozymes are widely exist in viruses, bacteria, and lower eukaryotes. At present, seven types have been recognized, which are Type I introns, Type II introns, RnaseP, hammerhead ribozymes (HH), hairpin ribozymes, *Neurospora varkud* Satellite (*VS*) ribozymes, and hepatitis delta virus (HDV) ribozymes (Deng et al., 2022). Among them, the HH consists of only 30–50 nt and requires divalent metal ions such as Mg^2+^ to participate in the catalytic process, and the specific cleavage site is at the 3′ end of triplet NUX (N stands for any base; X stands for C, U, A, but not G; Teplova et al., 2020). It is the only ribozyme that has been successfully applied to filamentous fungi and *A*. *giganteus* is the first reported case, with seven different designed hammerhead ribozymes that could reduce the beta-glucuronidase (*uidA*) gene expression by up to 100% (Mueller et al., 2006). Compared with other gene silencing strategies, ribozymes possess unique properties such as smaller in molecular weight, more flexible rational design and relative stability in organisms. Therefore, ribozymes can also be used in combination with other techniques such as CRISPR, where Gao et al. fused a hammerhead-type ribozyme and a hepatitis delta virus ribozyme sequences flanking the sgRNA expression plasmid, and the resulting sgRNA then was released by activities of these two ribozyme more efficient and accurate (Gao and Zhao, 2014). Moreover, the heritability of ribozyme is not stable, and the necessary cofactors should meet the need for intracellular catalysis without imposing a harmful burden on cells. In addition, the efficiency of biological action on target mRNA requires to improve and appropriately modify ribozymes to prevent degradation (Torres-Martinez and Ruiz-Vazquez, 2017; Wang et al., 2017).

## 3. Transformation methods

Transformation refers to the entry of foreign genetic biomolecules carrying modification information into the recipient cell, which may hampered by varying extents of the rigid cell wall and the multicellular structure of fungi. Allowing exogenous biomolecules (including DNA/RNA fragments, circular or linearized plasmids, and ribonucleoproteins) to pass through the cell wall and into as many as recipient cells are keys to breaking the bottleneck of low transformation efficiency of fungi. Currently, several proven transformation methods have been developed for *Aspergillus* spp., including Polyethylene glycol (PEG) Mediated Transformation (PMT) of protoplasts, *Agrobacterium tumefaciens* Mediated Transformation (AMT), Electroporation (EP), and Biolistic Transformation (BT). For a specialized review that focuses on transformation methods with filamentous fungi, the readers are directed to Yue et al. (2021). In addition, there are some physical methods, such as agitation with glass beads, vacuum infiltration, Shock Wave Mediated Transformation (SWMT), etc., which are relatively inefficient but can improve the shortcomings of the universal methods and play an auxiliary role (Table 3; Rivera et al., 2014; Li et al., 2017). The rate of these transformation approaches depends on the technique and the target species. However, some effective and simple methods that established in mammalian cell researches are not suitable for fungi, such as viral vector mediated transformation, nanomaterial mediated transformation, and liposome mediated transformation (Wang, 2021). Combining transformation technologies and genome editing technologies is expected to realize the best modification of fungal genomes.

**Table 3:** Systematic comparison of transformation methods.

Method	Recipient cells	Principles	Key points	Advantages	Disadvantages	Source
PEG mediated transformation (PMT)	Protoplasts	The receptive cells (protoplasts) absorb foreign DNA precipitated with divalent calcium ions on the membrane surface under the action of PEG, and the cell membrane will release lipids to reveal pores and facilitate the entry of foreign nucleic acids after short heat shocks	Preparation and regeneration of protoplasts; Appropriate selection of PEG, temperature and osmotic buffer; Cell permeability inducers can be added	Not necessary special vectors and bacteria intermediary	The preparation of protoplasts is cumbersome and there are no uniform standards due to variable proportion and specific components of cell wall for different species and even within the same individual at different stages of development; Not all fungi are suitable for the preparation of protoplasts, limited to specific species and types	Case et al. (1979)
*Agrobacterium* Mediated Transformation (AMT)	Conidia, mycelia, fruiting bodies, etc.	*A*. *tumefaciens* integrates foreign genes between the transfer DNA (T-DNA) left and right border repeats of the binary vector into the host through the virulence region of the T-vector, DNA transfer is achieved during co-cultivation of *A*. *tumefaciens* with the fungus	Integration of foreign genes by *A*. *tumefaciens*, pay attention to Appropriate concentration of inducer AS, temperature and time of co-culture, ratio of recipient cells to *A*. *tumefaciema*, etc.	The types of receptor cells is not limited; Efficient, more single copy integration and easier to produce stable genetic transformants	Time-consuming and requires tedious optimization of various factors during co-cultivation; The T-DNA inserted into genome lacks integrity and thus difficult to identify the insertion sites; It is hard to obtain enough mutants for high-throughput screening	Bundock et al. (1995)
Electroporation (EP)	Protoplasts or spores	Electric pulses induce membrane permeabilization providing a local driving force for ionic and molecular transport through the pores	Selection and control of electric field condition parameters depend on types of cells, such as the capacitance, the pulse duration and frequency; and Pay attention to low temperature heat dissipation	Can be applied to any fungi *in vivo* or frozen; Efficient and easily optimized protocols; High repeatability; and Multiple copy integration is possible.	Depends on the electrophysiological characteristics of the fungus; Requires additional expensive instrumentation; and Raises the rate of cellular death.	Alagui et al. (1989)
Biolistic Transformation (BT), also known as particle bombardment and microprojectile bombardment	Any cell types or species	DNA are coated with small tungsten or gold particles and accelerated to penetrate the cell wall at high velocity	Metal particles packaging for exogenous nucleic acids and the instrumental settings	Not limited to cell types or species and independent of the physiological properties of the fungi; Transformation with multiple transgenes is possible; Foreign genes can be transformed instantaneously in the cytoplasm without being integrated into the genome	DNA can be damaged; Produces multiple copies of introduced genes; The projectile preparation is complex; Low efficiency; and Expensive.	Klein et al. (1987)
Agitation with glass beads	Yeast, and few species with thin cell walls	Agitation of the fungal cells with glass beads in the presence of carrier and plasmid DNA allows DNA absorption	The nature of recipient cells, and the effect of agitation amplitude on cell activity	Simple, fast, cheap, and safe protocol	Cells require osmotic support and may cause cell disruption; Genome randomly assigned; Low transformation efficiency	Costanzo and Fox (1988)
Vacuum infiltration	Certain type of fungi with low sporulation rate	Vacuum generates a negative atmospheric pressure that causes the air spaces between the cells to decrease allowing the infiltration of *Agrobacterium*	Mediating the incorporation of *Agrobacterium* for fungi transformation	Can be used for fungi that produce little or no spores	Requires the use of bacteria which may have unwanted consequences	Bechtold et al. (1993)
Shock wave mediated transformation (SWMT)	Conidia	Acoustic cavitation changes the permeability of the membrane facilitating the absorption of DNA	Determination of Shock wave parameters (frequency, energy, voltage, and number of shock waves)	Simple, safe, cheap, and good reproducibility; High efficiency and may be applicable to species that have never been transformed; Uniform parameters can be used to transform diverse species of fungi	Relatively high cost of the shock wave source; Expertise in shock wave physics required; Will destroy a certain amount of DNA, but the tandem shock wave will enhance the transformation and make up for the loss of DNA to some extent	Magana-Ortiz et al. (2013)

## 4. Challenges and solutions to gene editing technology

The main processes of gene editing technology include the transference of foreign target DNA fragments into the recipient cell nucleus, correct integration into the genome and efficient expression of target genes. Currently, target fragments are usually generated in the form of linear PCR cassettes, which are available mainly through two systems. One is the split-marker PCR system that requires two DNA products containing overlapping fragments of a selective marker. Another is the fusion PCR system that requires fusing three PCR products, two for the HR in the genomic DNA flaking a selection region and the other for a selective marker and/or the replaceable promoter (Yon and Fried, 1989; Szewczyk et al., 2006). It is also possible to use plasmids to carry fragments of interest, and both linear and circular cassettes are suitable for transformation. But more importantly, there are still great challenges for the correct homologous recombination of foreign DNA sequences into the genome and the efficient expression of target genes.

### 4.1. Improve the efficiency of homologous recombination

**Figure 6:**
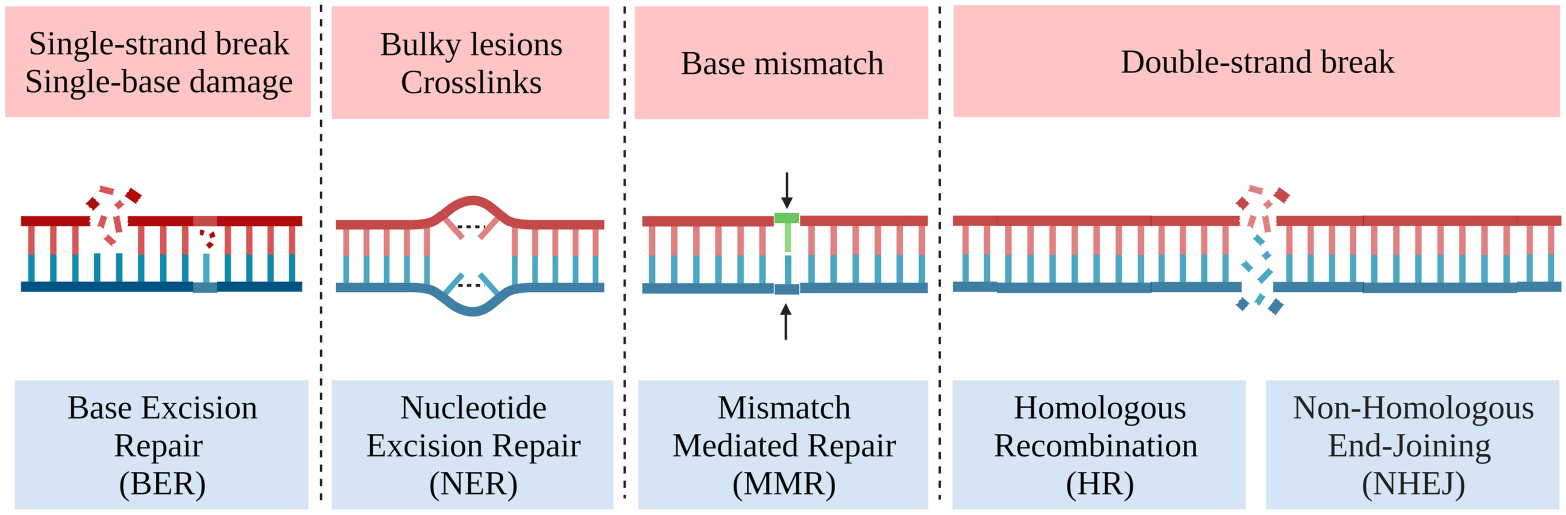
Different types of DNA damage and repair mechanisms.

Genetic damage and repair occur in cells at any time, accordingly, there are a variety of repair systems for different DNA damage (Figure 6; Ciccia and Elledge, 2010). The Double-Strand Break Repair (DSBR) systems in eukaryotic cells include the Non-Homologous End Joining (NHEJ), which is simpler but prone to random insertions and deletions, and Homology Directed Repair (HDR) that requires the presence of a homologous template to activate, which is necessary for the precise integration of DNA fragments into specific genomic loci (Szostak et al., 1983; Scully et al., 2019). However, eukaryotic microorganisms have a complex genetic background and inherently less HDR efficiency, and at least two generations are required to obtain stable homozygotes. All these are important reasons limiting the application of gene editing technology and solutions always from the perspective of improving HDR efficiency.

#### 4.1.1. Physicochemical methods

Firstly, synchronize the cell cycle. In wild-type cells, the HDR functions only in the S and G2 phases of the cycle, while NHEJ can occur throughout all the cell cycle phases (Deriano and Roth, 2013). Usually, the G1/G0 phase of cells has the DNA content of diploid cells (2 N), while the G2/M phase has the DNA content of tetraploid cells (4 N), while the DNA content of the S phase is between diploid and tetraploid. The detection of intracellular DNA content by insertive nucleic acid fluorescent dye combined with flow cytometry can screen out S phase cells. Furthermore, some small molecules, such as hydroxyurea (HU) and nocodazole, can prevent the reduction of nucleotides to deoxynucleotides and reversibly inhibit DNA synthesis in the S phase without affecting other cell cycle operations (Rosenkranz and Levy, 1965; Sinclair and Morton, 1965). But these cell cycle synchronization methods inevitably produce abnormal cell division and growth.

Alternatively, adding enhancers or inhibitors targeting DSBR components. The main components involved in NHEJ including KU70-KU80 heterodimer that binds to the cleaved double-strand DNA end to recruit other proteins and pull the two separated DNA segments together. DNA-PK_CS_ and its substrate Artemis, which are responsible for hydrolyzing the terminal single-stranded region at the end of DNA to create an effective substrate for ligase. DNA ligase IV together with XRCC4 catalyzes the formation of a new linkage between the DNA termini that have been processed by Artemis (Scully et al., 2019). Previous studies suggested that various small molecule compounds that repress the NHEJ pathway can effectively enhance the efficiency of HDR, such as the W7 and chlorpromazine (inhibit the production of KU co-factor), the vanillin, Nu7026 and Nu7441 (inhibit DNA-PK_CS_ activity), and the most commonly used inhibitor Scr7 (inhibit DNA ligase IV activity; Ying et al., 2019). On the other hand, it has been reported that RS-1 acts as an enhancer and promotes the HDR efficiency of mammalian cells, but there have been no reports in fungi (Jayathilaka et al., 2008). However, it is worth noting that some molecules are cytotoxic and need to determine the threshold and range of toxic effects.

#### 4.1.2. Biotechnological methods

Successful HDR requires single or double homologous fragments between the donor and acceptor DNA, but base mismatches will seriously reduce its frequency, and thus the length of the flanking sequences is an important decisive factor of HDR efficiency. Different organisms require different homologous arm lengths, and the upper limit of homologous recombination efficiency can be achieved varies greatly (Fujitani et al., 1995). In yeast, 50–100 bp homologous sequences can accomplish gene replacement and achieve 50–100% homologous recombination efficiency (Takita et al., 1997), while filamentous fungi require at least 1 kb homologous region but the efficiency of correct homologous recombination is still below 30%. It is necessary to determine the optimal length based on the species of *Aspergillus*, the size of the inserted DNA fragment, and similar properties of the system.

Non-Homologous End Joining and HR are two different repair pathways but NHEJ dominants DSB repair, so inhibiting the function of essential factors (including KU70/KU80, DNA ligaseIV, and their homologs) in the NHEJ pathway would improve the efficiency of HDR. Ninomiya Y et al. reported that inactivation of the NHEJ pathway led to 100% homologous recombination efficiency in *N*. *crassa*, and then the approach was rapidly developed in other filamentous fungi (Ninomiya et al., 2004). Currently, it is widely used to construct engineered *Aspergillus* with convenient NHEJ defect background to achieve efficient further gene editing, such as *ΔKu70/Ku80* (including *A*. *nidulans*, *A*. *fumigatus*, *A*. *sojae*, *A*. *oryzae*, *A*. *niger*, *A*. *parasiticus*, *A*. *flavus*, *A*. *chevalieri* var. *intermedius*, and *A*. *westerdijkiae*), and *ΔligD* (including *A*. *oryzae* and *A*. *luchuensis*; Ying et al., 2019; Madhavan et al., 2022). In addition, in strains loss of NHEJ ability, the integration efficiency is highly dependent on the targeted gene locus.

### 4.2. Efficient expression of foreign genes

The expression of heterologous genes can be limited in transcription, post-transcription, and translation levels. Several genetic strategies have been applied to reduce the expression constraints and enhance gene functionality.

Theoretically, increasing the copy number of the target gene can improve its transcription level, but it also depends largely on the integration place of the expression cassette in the genome (Graessle et al., 1997). AT-rich sequences in the coding regions act as internal polyadenylation sequences and produce incomplete transcripts that will limit heterologous gene expression at the transcriptional level, replacing an AT-rich sequence with a more GC-rich sequence can overcome the premature termination of transcription (Roberts et al., 1992). In addition, the presence of introns in eukaryotic systems can effectively improve mRNA stability. Deletion of introns in *A*. *niger* almost completely inhibited endogenous acid lipase expression (Zhu et al., 2020), and artificially adding introns to heterologous protein genes may be another way to improve mRNA stability (Deckers et al., 2020).

A more feasible method is the modification of the pre-transcriptional level, using strong promoters or optimized codons in desired gene cassettes for integration. Since the original pathway regulation is interactive and the expression of a gene is coordinated by both global and pathway specific multiple systems, selecting the appropriate promoter can be difficult but necessary. So far, a series of strong promoters are divided into constituent promoters and inducible promoters, which were developed to induce the expression of heterologous genes. The constituent promoters mainly include translation-elongation factor 1α (*tef1*), glyceraldehyde-3-phosphate dehydrogenase (*gpdA*), pyruvate kinase (*pkiA*), phosphoglycerate kinase (*pgkA*), etc. The inducible promoters mainly include xylose (*xlnA*), glucoamylase alpha (*glaA*), alpha-amylase (*amyA*), xylanase (*exlA*), cellobiohydrolase I (*cbh1*), heat shock protein (*hsp70*), etc. (Cazier and Blazeck, 2021; Garrigues et al., 2021; Zhuo et al., 2021). In addition, the alterations in 5’UTR can be used in combination with various strong promoters to enhance the expression of heterologous genes (Cao et al., 2021).

Genes show a nonrandom usage of synonymous codons and well-expressed genes are highly biased toward a subset of the present synonymous codons, significant heterogeneity in the codon usage exists among genes within species and the grade of codon bias is positively correlated with gene expression (Karlin and Mrazek, 2000). Therefore, codon optimization is also an effective method for attaining faster translation rates and higher accuracy, genes can be manipulated with rare codons replaced by their optimal synonyms and then reconstructed by overlap extension of synthetic oligonucleotides, improving the expression levels of heterologous genes (Koushki et al., 2011; Tanaka et al., 2014).

### 4.3. Screening for correct transformants

Effective selective markers are critical for molecular manipulation, including drug resistance genes, autotrophic or nutritional genes, and visual distinction reporters, which are all used for screening positive filamentous fungi (Table 4; Jin et al., 2021).

**Table 4:** Commonly used selective markers in *Aspergillus* spp.

Type	Name	Origin	Selection system	Product
Auxotroph marker	*ade1*	*Phanerochaete chrysosporium*	Adenine prototrophy	Phosphoribosylaminoimidazole-Succinocarboxamidesynthase
	*ade5*	*Nostoc commune*	Adenine prototrophy	Phosphoribosylaminoimidazole-succinocarboxamidesynthase
	*agaA*	*Aspergillus niger*	Ornithine prototrophy	Arginine hydrolase
	*argB*	*Aspergillus nidulans*	Arginine prototrophy	Acetylglutamate kinase
	*am*	*Neurospora crassa*	Glutamic acid prototrophy	Glutamate dehydrogenase
	*met2*	*Ascobolus immerses*	Methionine prototrophy	Hyperserine-O-acetyltransferase
	*pyrG*	*Aspergillus oryzae*	Pyrithiamine prototrophy	Orotidine-5′-phosphate decarboxylase
	*trpC*	*Aspergillus niger*	Tryptophan prototrophy	Indole-3-phosphate glycerol synthase
	*ura5*	*Podospora anserine*	Uracil prototrophy	Orotidine-5′-pyrophosphorylase
Drug (antibiotic) resistance marker	*bar*	*Streptomyces hygroscopicus*	Phosphinothricin resistance	Bialaphos acetyltransferase
	*benA*	*Neurospora crassa*	Phenylbacterim resistance	Beta tubulin mutant
	*ble*	*Escherichia coli*	Bleomycin resistance	Bleomycin binding protein
	*hygB*	*Escherichia coli*	Hygromycin B resistance	Hygromycin B phosphotransferase
	*neo*	*Escherichia coli*	Neomycin (G418) resistance	Neomycin phosphotransferase
	*oliC3*	*Aspergillus nidulans*	Oligomycin resistance	Oligomycin resistant protein
	*qa-2*	*Neurospora crassa*	Quinic acid utilization	Catabolic dehydrogenquinidase
Functional product marker	*acuD*	*Aspergillus nidulans*	Acetic acid utilization	Isocitratlyase
	*amdS*	*Aspergillus nidulans*	Acetamide utilization	Acetamidase
	*inl*	*Neurospora crassa*	Inositol utilization	Inositol phosphate synthase
	*lacZ*	*Escherichia coli*	Spore color selection (white → blue)	Beta-galactosidase
	*niaD*	*Aspergillus oryzae*	Nitrate utilization	Nitratase
	*nit2*	*Neurospora crassa*	Nitrogen source utilization	Nitrogen metabolite repressor regulation
	*pal*	*Rhodosporidium toruloides*	Phenylalanine	Phenylalanine dehydrogenase
	*pro*	*Aspergillus nidulans*	Proline utilization	Proline metabolizing enzyme
	*qutE*	*Aspergillus nidulans*	Quinic acid utilization	3-dehydrogenquinic acid decomposing enzyme
	*sC*	*Aspergillus nidulans*	Nitrate utilization	ATP sulfatase
	*yA*	*Aspergillus nidulans*	Spore color selection (yellow → green)	

Drug (antibiotic) resistance genes are generally used as dominant selection markers in species that are no endogenous drug resistance. For example, the *A*. *flavus* NRRL 3357 is resistant to hygromycin, so *hygB* gene cannot be used as the marker gene, while the *ble* and *pyr* genes have been successfully used for *A*. *flavus* transformation. The other limitation is that antibiotics are usually expensive, and there are concerns about issues with the contamination of genetically modified organisms. Auxotrophic genes encode essential proteins for the biosynthesis of necessary nutrients, among them, the *pyrG* gene is the most widely used marker. PyrG is involved in uridine biosynthesis and is also a target for the antimetabolite 5-fluoroorotic acid (5-FOA). The *pyrG* deficient mutants are resistant to 5-FOA, meanwhile, it could not grow in media without uridine or uracil because of the loss of ability to synthesize uracil autologously. Therefore, it can show a two-way screening effect and distinguish the wild type and mutant from two perspectives (Wang et al., 2022). Fluorescent signal genes encode fluorescent proteins that can be visually distinguished, such as enhanced Green Fluorescent Protein (eGFP) and *Discosoma* sp. Red fluorescent protein (DsRed; Chen et al., 2019). It is relatively simple to operate but requires special observation instruments. In addition, phenotypic differences, such as pigment production, colony color or traits between wild-type strains and mutant strains can be used to achieve marker free screening of mutants, albeit only in individual samples. For *A*. *fumigatus*, the mutants of expected genomic alteration can be screened by the colorless (albino; Fuller et al., 2015). In *A*. *carbonarius*, the yellow conidia mutants can also be clearly distinguished from the wild type of the black conidia (Weyda et al., 2017).

Notably, the combination of multiple selection markers can greatly reduce false positives and enable the construction of multi-gene mutants or complemented strains conveniently. However, there are still fewer available selection markers for continuous genetic replacement, and may not be suitable for any strain. The solution is to combine recombinase and reuse marker genes, which allows the same selection gene for screening the positive clones. Zhang et al. constructed a one-step unmarked genetic modification by the Cre-*lox*P based CRISPR-Cas9 system, the target gene is knocked out through the CRISPR-Cas9 system, and Cre recombinase is induced by light and then deletes selection markers, which ensures that the same marker can be used for future genetic manipulation (Zhang et al., 2016). This one-step unmarked genetic modification system has been successfully applied in *Hypocrea jecorina* Qm6a, *N*. *crassa*, *A*. *niger*, *Fusarium graminearum*, *Metarhizium anisopliae*, etc. (Connolly et al., 2018; Enghiad et al., 2021; Rozhkova and Kislitsin, 2021).

## 5. Conclusion and future directions

*Aspergillus* with powerful and attractive ability to synthesize, modified, and secrete various metabolites. Filamentous fungi express more than 50% of industrial enzymes, in which *Aspergillus* plays a vital role in expressing these industrial enzymes (Arnau et al., 2020). Compared to bacteria and yeasts, *Aspergillus* performs polarized cell growth, where hyphal tip secretes proteins intensively and efficiently, reaching 10–1,000-fold of bacterial, yeast, or mammalian cells. On the other hand, *Aspergillus* has a variety of enzymes that can decompose and utilize a variety of carbon and nitrogen sources, as cell factories which have more diverse fermentation substrates, and extracellular proteins are easier to purify. But the development of filamentous fungal expression platforms is much more complex, time-consuming, and needs extensive development. At present, *A*. *niger* and *A*. *oryzae* have been used as mature cell factories to produce enzymes, recombinant proteins, antibiotics, organic acids, polyunsaturated fatty acids, nutrients, etc. in industrialization, species of *Aspergillus* can decompose plastics and pollutants, it has been widely used in the pharmaceutical, agricultural and food industries, as well as the environmental field, safely, efficiently, and economically (Ying et al., 2019; Li et al., 2022). Even mycotoxins synthesis, which is considered harmful and regulated comprehensively through complex pathways such as anabolism and catabolism, can be controlled in the cascade of external environmental factors (Gao et al., 2021), the knockdown of key genes in mycotoxin biosynthesis gene clusters or cross-regulatory pathways can prevent and control harmful mycotoxin synthesis from the source. Overexpression of specific genes can activate silencing pathways and may discover more useful secondary metabolites (Yuan et al., 2022). Therefore, the study of *Aspergillus* inevitably needs to go deep into the genetic level, the exploration of mechanisms and modification of gene functions can be realized through gene editing technologies by pathways reconstruction or activating silent pathways. Gene manipulation systems require vector construction, transformation, and genome editing, with the ultimate goal of achieving the expression of desirable traits and stable inheritance. But opportunities and challenges coexist, firstly, *Aspergillus* genome background is limited, and up to now the available gene information is still lacking except for model strains. For transformation, compared with other microorganisms such as yeast and bacteria, the transfer of exogenous vectors is blocked by rigid cell wall and complex mycelial structure. From the perspective of gene fragment integration, due to the intrinsic ploidy, propagation, and inefficient homologous recombination, it has not been as efficient as desired, and so far, no industrial strains built using the CRISPR technology have been used commercially. In conclusion, the search for a versatile, effective and stable genetic tool suitable for *Aspergillus* still confronted with many challenges.

Traditional gene-level gene editing strategies have certain technological barriers, such as the design process being complex and relatively costly and time-consuming, the off-target rate being high, only a single gene can be modified at a time, and only simple gene knockout or knockin can be performed. Further transcription-level editing can enable genome-scale knockout screening, genome architecture engineering, and RNA editing. The advent of new technologies, such as CRISPR-Cas, provides brand new ideas and greater operable space. The selective uniting of multiple gene editing technologies and the combination with efficient expression systems have significantly improved the precision and accuracy of biotechnology and applications, and the scope of function is evolving from single to multi-gene and from low to high throughput (Yilmaz, 2021; Antony et al., 2022). In the future, other types of genetic modifications may be achieved, such as gene motivation and gene interference with a controlled degree of expression, exploring more orthologues of functional enzymes within organisms, as well as cross-species gene mapping. Under the premise of ethics, gene editing technology is bound to become a powerful tool for humans.

## Author contributions

JG and ZL: conceptualization and formal analysis. JG and ZZ: writing—original draft preparation. JG and HL: writing—review and editing. ZL and HK: supervision and validation. All authors contributed to the article and approved the submitted version.

## Funding

This work was supported by the National Natural Science Foundation of China (No. 32172170) and Graduate Independent Innovation Research Foundation of China Agricultural University (No. 2022TC160).

## Conflict of interest

The authors declare that the research was conducted in the absence of any commercial or financial relationships that could be construed as a potential conflict of interest.

## Publisher’s note

All claims expressed in this article are solely those of the authors and do not necessarily represent those of their affiliated organizations, or those of the publisher, the editors and the reviewers. Any product that may be evaluated in this article, or claim that may be made by its manufacturer, is not guaranteed or endorsed by the publisher.
